# Effects of negative life events on depression in middle school students: The chain-mediating roles of rumination and perceived social support

**DOI:** 10.3389/fpsyg.2022.781274

**Published:** 2022-08-12

**Authors:** Hui Xia, Xuexue Han, Jing Cheng, Debiao Liu, Yili Wu, Yan Liu

**Affiliations:** ^1^School of Mental Health, Jining Medical University, Jining, China; ^2^Cheeloo College of Medicine, Shandong University, Jinan, China; ^3^School of Mental Health and The Affiliated Kangning Hospital, Wenzhou Medical University, Wenzhou, China; ^4^Key Laboratory of Alzheimer’s Disease of Zhejiang Province, Institute of Aging, Zhejiang Provincial Clinical Research Center for Mental Disorders, Wenzhou, China; ^5^Oujiang Laboratory, Zhejiang Lab for Regenerative Medicine, Vision and Brain Health, Wenzhou, China; ^6^Shandong Key Laboratory of Behavioral Medicine, School of Mental Health, Jining Medical University, Jining, China; ^7^Shandong Collaborative Innovation Center for Diagnosis & Treatment & Behavioral Interventions of Mental Disorders, Institute of Mental Health, Jining Medical University, Jining, China; ^8^Center of Evidence-Based Medicine, Jining Medical University, Jining, China

**Keywords:** middle school student, negative life events, depression, rumination, perceived social support, chain mediation

## Abstract

**Background:**

Negative life events in middle school students have a significant impact on depression. However, the mechanism of this association is not fully understood. This study used rumination and perceived social support as mediating variables to explore the influence of negative life events on depression.

**Materials and methods:**

Due to the COVID-19 pandemic and social distancing, a convenient sampling method was adopted to collect information about middle school students in Shandong Province by means of online questionnaire. Adolescent Self-Rating Life Events Check List, Ruminative Responses Scale, Perceived Social Support Scale and Children’s Depression Inventory were used. Descriptive statistics and correlation analysis were conducted for four variables of middle school students, including life events, depression, rumination thinking and perceived social support, and the chain mediated effect was tested by using process plug-in. All statistically analysis was conducted by SPSS 23.0.

**Results:**

493 middle school students (16.7000 ± 0.9500 years) including 343 female students (69.6000%) from Shandong Province recruited. Results showed that the total effect between life events and depression was significant (effect = 0.2535, 95%CI: 0.2146, 0.2924). The total indirect effect was significant (effect = 0.1700, 95%CI: 0.1349, 0.2072). The indirect effect was significant (effect = 0.0988, 95%CI: 0.0741, 0.1252) with rumination as the mediating variable. The indirect effect of pathway with perceived social support as the mediating variable was significant (effect = 0.0476, 95%CI: 0.0295, 0.0674). The indirect effect of pathway with rumination and perceived social support as mediating variables was also significant (effect = 0.0236, 95%CI: 0.0147, 0.0339).

**Conclusion:**

This study indicated that ruminant thinking and perceived social support had a significant chain mediating effect on adolescents’ life events and depression. Life events can not only directly affect depressive emotions, but also indirectly affect depressive emotions by affecting ruminant thinking and perceived social support. The results of this study not only provide new directions for the relationship between life events and depression, but also provide possible approaches for future prevention and intervention of depression in middle school students.

## Background

Adolescence is a special stage of adolescent development. At this stage, adolescents are more likely to have various mental health problems, especially depression (Hards et al., 2020). Depression is mainly characterized by persistent low mood, loss of interest, self-accusation, inattention, sleep disorder, somatic symptoms, and other clinical manifestations (Rice et al., 2019). The meta-analysis showed that the prevalence of depression in Chinese junior high school students was 16.2000%, while that in senior high school students was 22.1000% (Li et al., 2016). Depression has severe impacts on adolescents’ physical and mental health, academic performance, and social functions and even causes non-suicidal self-mutilation and suicide which brings a heavy burden to the society (Steinberg, 2004; Zhou et al., 2012; Li et al., 2016). Negative life events indirectly lead to depression among adolescents (Xia and Ma, 2018; Jiang et al., 2019). It was reported that rumination has been found to mediate the relationship between negative life events and depressive symptoms (Łosiak et al., 2019). In addition, perceived social support also plays an important role in moderating effect between negative stressful life events and depression and acts as a buffer against stressful events (Ruscio et al., 2015; Miloseva et al., 2017). However, previous studies were limited to a single mediating model. To confirm the complex psychological process between negative life events and depression, we ventured to hypothesize that rumination plays an intermediary role in life events and depression by influencing perceived social support, or perceived social support plays a mediating role in life events and depression by influencing rumination. Therefore, this study is a quantitative study based on questionnaire data.

### Relationship between negative life events and depression in middle school students

Middle school students have to face all kinds of pressure, such as academic evaluation, coordination with teachers, classmates, friends and relatives, and so on. Major negative life events may cause stress state changes, promoting a series of physiological and psychological changes, which will lead to inefficient learning, reduced life adaptability, and even depression. According to the diathesis-stress theory of depression, stressor caused by life events and bad thinking mode such as negative reasoning style and ruminant are risk factors for depression, which activates the individual’s potential predisposition (Monroe and Simons, 1991). Negative life events can effectively predict the occurrence of depression (Phillips et al., 2015). The total score of negative life events of middle school students was positively correlated with depression; that is, the more negative life events experienced, the more likely for middle school students to have depression (Miao, 2020).

### Mediating role of rumination

A ruminative response style refers to the negative emotions that individuals repeatedly focus on themselves. People with this reaction style spend a lot of time focusing on their negative emotions (Treynor et al., 2003), reflecting on their causes, and worrying about the consequences, rather than taking action to change the situation. The research has also shown that rumination can exacerbate depression by amplifying negative emotional states and immersing in them, reducing the ability to respond to emergencies and the willingness to engage in pleasurable activities (Watkins and Roberts, 2020).

As rumination is a typical negative information processing process, it plays an important role in the influence of negative life events on mood. The study shows that rumination was found to mediate the relationship between negative life events and suicidal ideation (Wang et al., 2020). In the face of negative events, individuals tend to ruminate on negative events and negative emotions, which is an important psychological mechanism. It has been demonstrated that interest in rousing painful emotions and rethinking uncertain future events increased with prolonged immersion in the pain of a bereavement, which leads to a decrease in the ability to adapt to bereavement and related avoidance behavior (Eisma et al., 2020). In addition, the study also shows that people who are prone to ruminating tend to react negatively to stressful events and express a pessimistic view of positive future events (Qiao et al., 2013). In addition, Louisa et al., taking adolescents as an example, confirmed that self-reported to stressful life events are correlated with increased rumination activities (Michl et al., 2013).

Rumination can lead to depression in a number of ways. Rumination was found to be a strong predictor of depressive symptoms in a study of adolescents with internalization disorders. Persistent focus on negative things can lead to behavioral and emotional abnormalities that can lead to depression (Jandrić et al., 2021). According to the theory of the reactive depression model, a ruminant response style individual is more likely to be depressed. Rumination can enhance depression influence on thinking. Depressed people will activate the negative thoughts to reflect on the current situation, and rumination can make thought stagnation in the current moment and not to think about how to effectively solve the problem. Finally, rumination can also inhibit instrumental behavior, leading to increased pressure (Nolen-Hoeksema, 1991). It also demonstrated the mediating role of rumination as a longitudinal association between self-reported to stressful life events and symptoms of depression and anxiety (Michl et al., 2013). In conclusion, there is a relationship between negative life events and rumination and between rumination and depression. It turns out that the life events we suffer are associated with an increase in rumination activity, which in turn is linked to depression. Therefore, it is reasonable to hypothesize that rumination is indirectly related to depression through life events.

### Mediating role of perceived social support

Social support is a series of support that a person receives through the exchange of information, emotional interaction, and material help with others (Tariq et al., 2020). Perceived social support refers to the material, psychological, and other support that can be truly perceived by friends, family, and society when individual needs support (Grey et al., 2020). Some literature proves that perceived social support is only moderately correlated with social support behavior (Lakey et al., 2010). The buffer model of social support believes that social support can reduce the impact of stressful events on people’s emotions and behaviors and make people better adapt to the environment (Cohen and Wills, 1985). Liu found that social support plays a mediating role in the relationship between negative life events and adjustment. The moderating and mediating factors of the development of depression have been the focus of the research. Previous studies have suggested that social support plays a protective role in the relationship between life events and depression, reducing the influence of negative life events on individuals (Gariépy et al., 2016; Liu et al., 2020). Yang et al. found that perceived social support played a powerful role in reducing stress and was also found to be effective in reducing depressive symptoms (Yang, 2006). Few literatures have explored the mediating role of perceived social support in the relationship between life events and depression in adolescents. However, Liu et al. confirmed our hypothesis that perceived social support plays a mediating role in the association between life events and depression in adolescents (Birkeland et al., 2021). In summary, we can know that when adolescents are hit by life events, if they get more social support or perceive more social support, it is beneficial to alleviate the symptoms of depression.

### Relationship between rumination and perceived social support

Given that rumination and perceived social support are associated with depression, we took the bold step of linking the two concepts together. In fact, the research has shown that people with a ruminative style will repeatedly recall negative, irrational thoughts related to the trauma during a traumatic event, but sharing ruminant thoughts with a supportive person can help the individual reconceptualize the traumatic event and ease concerns related to the current trauma (Liu et al., 2020). In addition, a research has confirmed that rumination is a risk factor for depressive symptoms in Australian women (Turner and Mclaren, 2011). However, the study did not find a relationship between social support and rumination and depressive symptoms (Turner and Mclaren, 2011). In one study, improving effective communication in supportive relationships can help individuals reduce self-centered rumination, which may be beneficial in reducing depressive symptoms (Ames-Sikora et al., 2017). To further clarify the relationship between rumination, perceived social support, and depression, we hypothesized that rumination could be alleviated by perceived social support.

The purpose of this study is to explore the relationship between adolescent life events and depression and to explore the possible pathways of this relationship in terms of both rumination and perceived social support. We assumed that the increase of life events by ruminant thinking and perceived social support are indirectly associated with depression with four indirect effects: (1) life events → rumination → depression, (2) life events → perceived social support → depression, (3) life events → rumination → perceived social support → depression, and (4) life events → perceived social support → rumination → depression.

## Materials and methods

### Participants

Due to the COVID-19 pandemic and social distancing, a convenient sampling method was adopted to collect information about middle school students in Shandong Province, China, by means of online questionnaire. The questionnaire was distributed in the spring from 15 March to 6 April 2020, and the collection time was 23 days in total. We all got the consent and approval of the participants’ parents who answered the questionnaire, and most of the subjects filled out the questionnaire with the mobilization of school teachers. We collected 516 questionnaires (none of them had zero answers or refused to answer some questions), but only 493 questionnaires (including middle school students in Jinan and Heze, Shandong Province, China) were used in the end. Three criteria were excluded from these 23 questionnaires: the first was that the answer time was too long or too short, the second was that the age input seriously deviated from the middle school students’ age range, and the third was that there were a large number of consistent answers in one or more questionnaires. The above three situations are not included in the scope of the valid questionnaire (the inclusion criteria of this questionnaire are: first, the age should meet the requirements of middle school students; second, answer the questions carefully, and there is no consistent answer or regular answer in the whole article). Since the excluded questionnaire only accounts for 4.46% of the total questionnaires, there will be no sampling deviation in the probability, which will affect the normal distribution of the whole data.

### Measurements

#### Adolescent Self-Rating Life Events Checklist

It is composed of 27 items. The evaluation period of 3–12 months can be selected according to the research situation, and 12 months is selected as the evaluation period in this study. The scale includes six dimensions: interpersonal relationship (1, 2, 4, 15, 25), learning pressure (3, 9, 16, 18, 22), punishment (17, 18, 19, 20, 21, 23, 24), loss (12, 13, 14), health adaptation (5, 8, 11, 27), and other (6, 7, 23, 24). During the assessment, judge whether the project has occurred within the specified time (Liu et al., 1997a). If the event does not occur, it will be recorded as no impact; if it has, it is divided into five grades according to the impact of the event on itself (no impact: 1 point; slight impact: 2 points; moderate impact: 3 points; severe impact: 4 points; extremely severe impact: 5 points). The statistical indicators included the frequency of events and the amount of stress. The cumulative score of each event was the total stress amount (Liu et al., 1997c). It is found that the scale is suitable for middle school students for self-assessment, with good reliability and validity, and the scale has been revised and tested for many times (Monroe, 1982; Liu et al., 1997a). The study shows that the Cronbach’s coefficient α of the scale among Chinese adolescents was 0.8492, the split-half reliability coefficient was 0.8809, and the test–retest correlation coefficient was 0.6861. Factor analysis showed that the scale could be summarized by six factors (1474 adolescents aged 13–20 were selected as subjects, including 22 boys and 544 girls) (Liu et al., 1997b). The study shows that the Cronbach’s coefficient α of the scale among Chinese adolescents is 0.9200, the test–retest reliability is 0.7300, and the split-half reliability is 0.8500. Factor analysis showed that the scale could be summarized by five factors (the study involved 10566 middle school students in 10 cities across the country, 5036 boys and 5525 girls) (Xin and Yao, 2015), and its Cronbach coefficient in this study is 0.9410.

#### Children’s Depression Inventory

Children’s Depression Inventory (CDI) was used to assess depression in children and adolescents aged 7–17 in the past two weeks. The scale consists of 27 items, including five subscales: lack of happiness, negative emotions, low self-esteem, low efficiency, and interpersonal problems. Each item has three options to describe different degrees of depressive symptoms. The score is divided into three grades, which are 0–2 points, and the total score is 54 points. Nineteen points are regarded as the boundary points of depression symptoms. The higher the score, the higher the depression level (Wu et al., 2010). The scale is suitable for domestic primary and secondary school groups and has good reliability and validity. Its content is close to the life of primary and secondary school students and only needs the reading level of grade one. The study shows that the Cronbach’s coefficient α of the scale among Chinese adolescents was 0.8530, the reliability coefficients of each subscale ranged from 0.3480 to 0.6690, and the split-half reliability coefficient was 0.8240 (the study took 7161 middle school students in Nanjing and Jiangsu provinces of China as subjects, 3537 boys and 3624 girls) (Hong et al., 2012), and its Cronbach coefficient in this study is 0.8910.

#### Ruminative Responses Scale

It consists of 22 items, which are divided into three factors: symptomatic rumination (1, 2, 3, 4, 6, 8, 9, 14, 17, 19, 22), obsessive–compulsive thinking (5, 10, 11, 13, 15, 16, 18), and reflection (7, 12, 20, 21). The score was four grades, which were 1–4 (never-1, sometimes-2, often-3, always-4). The higher the score, the more serious rumination (Han and Yang, 2009). The research shows that the scale has good reliability and validity. The study shows that the Cronbach’s coefficient α of the scale among Chinese adolescents was 0.8800 (the study took 1311 middle school students in Yunnan and Guizhou provinces of China as subjects, 678 boys and 633 girls) (Yan and Zheng, 2006), and its Cronbach coefficient in this study is 0.9430.

#### Perceived Social Support Scale

Zimet et al. compiled a total of 12 projects, including family support (3, 4, 8, 11), friend support (6, 7, 9, 12), and other support (1, 2, 5, 10). The score of grade 7 was 1–7 (very disagree - 1, very agree - 7). According to the practice of Yan Biaobin and Zheng Xue, this study changed “leader, relative, and colleague” into “teacher, relative, and classmate” in the original scale (Tang et al., 2021). Relevant studies have shown that the perceived social support scale has good reliability and validity in Chinese adolescents, with a reliability coefficient of 0.9270 and a validity coefficient of KMO of 0.9270, the split-half reliability was 0.9050, the correlation coefficients of the subscale and the total scale of the perceived social support scale were 0.8860, 0.8870, and 0.9620 (*p* < 0.001), and the correlation coefficients of each item and each dimension were 0.6410, 0.7500, and 0.7500 (*p* < 0.001). The structural validity of the scale was high (the study took 489 middle school students in Guizhou Province as subjects, 220 boys and 269 girls) (Ye and Zhu, 2022), and its Cronbach coefficient in this study is 0.9420.

### Statistical analysis

The excel function of WPS 2019 was used to input and sort out the data, and SPSS 23.0 and process plug-in were used for data analysis. Demographic analysis, correlation analysis of main variables, multiple linear regression, and chain intermediary effect were carried out on the questionnaire (bootstrap samples 5000 times with a confidence interval of 95%).

## Results

### Descriptive analysis and correlation analysis

A total of 516 questionnaires were collected, 493 of which were effective, and the effective rate was 95.5400%. Among them, 150 were male (30.4000%) and 343 were female (69.6000%). The mean age was 16.7000 ± 0.9500 years old, ranging from 13 to 19 years. Among them, 473 subjects (95.9000%) were from Jinan, Shandong, China, and 20 subjects (4.1000%) were from Heze, Shandong, China. The grade distribution ranges from grade 1 of junior high school to grade 3 of senior high school, including 5 subjects in grade 1 of junior high school (1.0000%), 46 subjects in grade 2 of junior high school (9.3000%), 18 subjects in grade 3 of junior high school (3.7000%), 420 subjects in grade 2 of senior high school (85.2000%), and 4 subjects in grade 3 of senior high school (0.8000%).

Adolescent life events, rumination, perceived social support, and depression were analyzed by correlation analysis. Table 1 lists the mean, standard deviation, and correlation matrix of each major study variable. As can be seen from Table 1, life events are significantly positively correlated with rumination (*r* = 0.4350, *p* < 0.01), negatively correlated with perceived social support (*r* = −0.3390, *p* < 0.01), and positively correlated with depression (*r* = 0.5090, *p* < 0.01). There was a significant negative correlation between rumination and perceived social support (*r* = −0.3640, *p* < 0.01) and positively correlated with depression (*r* = 0.6850, *p* < 0.01). There was a significant negative correlation between perceived social support and depression (*r* = −0.6100, *p* < 0.01).

**TABLE 1:** Descriptive statistics of each variable and the correlation analysis results (*N* = 493) **p < 0.01.

Variables	Mean ± SD	Negative life events	Rumination	Perceived social support	Depression
Negative life events	44.0300 ± 15.3780	1			
Rumination	45.9200 ± 11.9400	0.4350**	1		
Perceived social support	61.5000 ± 13.8640	−0.3390**	−0.3640**	1	
Depression	12.9700 ± 7.8060	0.5090**	0.6850**	−0.6100**	1

### Mediating effect of rumination and perceived social support

**FIGURE 1:**
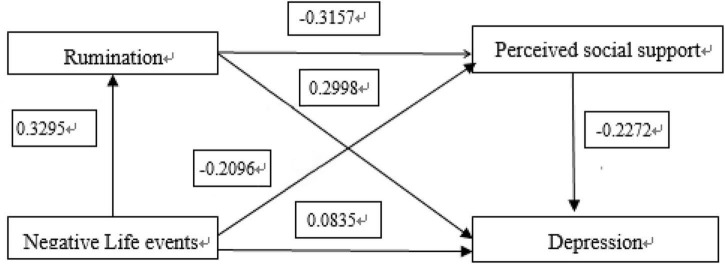
Ruminating and perceiving social support influences in the chain-mediated model of the relationship between negative life events and depression.

**FIGURE 2:**
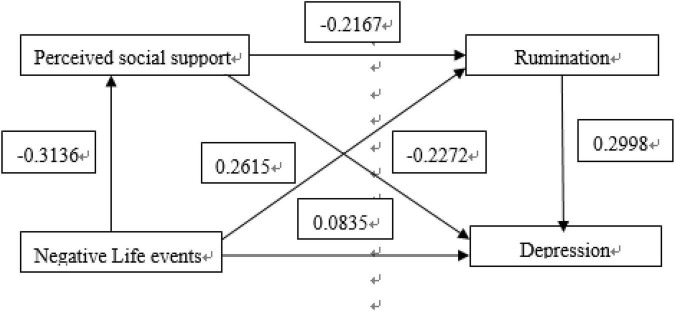
Perceiving social support and ruminating influences in the chain-mediated model of the relationship between negative life events and depression.

Model 6 of SPSS plug-in Process provided by Hayes (2013) was used (Bolin, 2014). Life events were taken as independent variable, depression as dependent variable, and ruminant and perceived social support as chained mediating variable. The path coefficient results are shown in Figures 1, 2.

Table 2 lists the results of multiple linear regression analysis of major research variables. It can be seen from Table 2 that life events can significantly positively predict rumination (β = 0.4240, *p* < 0.001) and depression (β = −0.1640, *p* < 0.001) and significantly negatively predict perceived social support (β = −0.2330, *p* < 0.001); ruminant thought was a significant negative predictor of perceived social support (β = −0.2720, *p* < 0.001) and significant positive predictor of depression (β = 0.4580, *p* < 0.001), while perceived social support was a significant negative predictor of depression (β = −0.4030, *p* < 0.001).

**TABLE 2:** Regression analysis results between variables.

Items	Regression equation	Integral fitting index			Significance of regression coefficient	
	Predictive variables	R	R^2^	*F*	β	ε
Rumination						
	Gender	0.4550	0.2070	25.4090	−0.0110	−0.2630
	Age				−0.0400	−0.5500
	City				0.0180	0.4140
	Grade				0.1690	2.2420*
	Life events				0.4240	10.4300***
Perceived social support						
	Gender	0.4490	0.2020	20.4650	0.1620	3.9670***
	Age				−0.0180	−0.2500
	City				0.0390	0.8800
	Grade				0.0460	0.6030
	Life events				−0.2330	−5.1450***
	Rumination				−0.2720	−5.9740***
Depression						
	Gender	0.8100	0.6560	132.4130	0.1080	3.9550***
	Age				−0.0620	−1.3090
	City				0.0260	0.8910
	Grade				0.0800	1.5990
	Life events				0.1640	5.3980***
	Rumination				0.4580	14.8080***
	Perceived social support				−0.4030	−13.5440***

*p < 0.05; ***p < 0.001.

Tables 3, 4 list the testing of the mediating effect of each major study variable. Bootstrap test further showed (Tables 3, 4) that the 95% confidence intervals of the three paths did not include 0, indicating that the three indirect effects reached significant levels. As can be seen from Table 3, the total effect between life events and depression is significant (effect = 0.2535, 95% CI: 0.2146, 0.2924). Meanwhile, the total indirect effect was significant (effect = 0.1700, 95% CI: 0.1355, 0.2070). The indirect effect was significant (effect = 0.0988, 95% CI: 0.0748, 0.1250) with rumination as the mediating variable. The indirect effect of pathway with perceived social support as the mediating variable was significant (effect = 0.0476, 95% CI: 0.0293, 0.0679). The indirect effect of pathway with rumination and perceived social support as mediating variables was also significant (effect = 0.0236, 95% CI: 0.0149, 0.0342). As can be seen from Table 4, the total effect between life events and depression is significant (effect = 0.2535, 95% CI: 0.2146, 0.2924). Meanwhile, the total indirect effect was significant (effect = 0.1700, 95% CI: 0.1351, 0.2065). The indirect effect was significant (effect = 0.0712, 95% CI: 0.0512, 0.0927) with rumination as the mediating variable. The indirect effect of pathway with perceived social support as the mediating variable was significant (effect = 0.0784, 95% CI: 0.0556, 0.1027). The indirect effect of pathway with rumination and perceived social support as mediating variables was also significant (effect = 0.0204, 95% CI: 0.0122, 0.0303). These results indicate that ruminant thinking and perceived social support have a significant chain-mediating effect on adolescents’ life events and depression. Life events can not only directly affect depressive emotions, but also indirectly affect depressive emotions by affecting ruminant thinking and perceived social support.

**TABLE 3:** Mediating effect test of rumination and perceived social support*.

Item	Effect size	Boot SE	Boot CI	
			Lower	Upper
Total effects	0.2535	0.0198	0.2146	0.2924
Direct effect	0.0835	0.0155	0.0531	0.1139
Total indirect effects	0.1700	0.0183	0.1355	0.2070
Indp1: LE→RU→DE	0.0988	0.0130	0.0748	0.1250
Indp2: LE→PSS→DE	0.0476	0.0097	0.0293	0.0679
Indp3: LE→RU→PSS→DE	0.0236	0.0050	0.0149	0.0342

*Indp, indirect path; LE, life events; RU, rumination; PSS, perceived social support; DE, depression; CI, confidence interval.

**TABLE 4:** Mediating effect test of perceived social support and rumination*.

Item	Effect Size	Boot SE	Boot CI	
			Lower	Upper
Total effects	0.2535	0.0198	0.2146	0.2924
Direct effect	0.0835	0.0155	0.0531	0.1139
Total indirect effects	0.1700	0.0183	0.1351	0.2065
Indp1: LE→PSS→DE	0.0712	0.0105	0.0512	0.0927
Indp2: LE→RU→DE	0.0784	0.0122	0.0556	0.1027
Indp3: LE→PSS→RU→DE	0.0204	0.0046	0.0122	0.0303

*Indp, indirect path; LE, life events; PSS, perceived social support; RU, rumination; DE, depression; CI, confidence interval.

## Discussion

There were pairwise correlations among life events, rumination, perceived social support, and depression. Rumination and perceived social support have multiple mediating effects on the relationship between life events and depression. Rumination and perceived social support can both affect the relationship between life events and depression of middle school students, alone or together.

This study reveals a significant negative correlation between adolescent negative life events and depression, which is consistent with previous studies (Liu et al., 2019). Negative life events are one of the environmental factors that affect mental health. The more negative life events teenagers encounter, the more likely they are to have depression (Xiu and Ji, 2018). Therefore, we can relieve the psychological pressure of teenagers, try to prevent the accumulation of negative life events, and do a good job of early warning and intervention of negative life events, so as to prevent the occurrence of depression.

Rumination plays a mediating role between life events and depression; that is, the more negative life events individuals encounter, the more their rumination will be aggravated, and the greater the possibility of depression will be. This is similar to the previous study that stressful life events increase the level of ruminant thinking in individuals with depressive experiences and thus increase the level of ruminant thinking in individuals with negative experiences (Wang et al., 2020). At the same time, high rumination thinking shows excessive attention to negative information. In this case, it is difficult to control or stop rumination thinking, and it will connect self with negative information, so that negative cognitive tendencies persist, and trigger and lead to depression (Arnarson et al., 2016). In addition, considering rumination as a bad thinking mode, we suggest that schools can carry out more psychological counseling services, provide more ways for adolescents to cope with negative life events, and reduce the adverse effects of rumination on adolescents.

Social support plays a mediating role between life events and depression, which verifies hypothesis 2 that the influence of excessive negative life events can be alleviated if adequate social support is obtained, which is consistent with the research results of Liu et al. (2020). Another study also showed that a lack of social support is associated with the onset and recurrence of depression (Paykel, 1994). It has also been shown that female adolescents with high perceived social support have lower levels of depression in response to stressful life events (Ouyang et al., 2021). It is well known that perceived social support has a wide range of health benefits. As a stress regulator, perceived social support can provide individuals with the psychological support needed to cope with stress, thereby reducing the harmful effects of accumulated negative life events on health (Chen et al., 2021). In the growth process of teenagers, they will inevitably encounter a variety of negative events and setbacks, and targeted social support may alleviate the adverse effects. Therefore, family members, teachers, friends, and other close relationship members can provide timely support and psychological counseling when teenagers face difficulties, which is of great significance to their healthy growth.

Rumination and perceived social support are chain-mediated model of life events and depression. Specifically, the life events through rumination and perceived social support indirectly associated with depression, namely that when individuals suffering too much negative life events exacerbate ruminant thinking level, will affect the individual self-adjusting ability and problem-solving ability, again by weakening the individual’s ability to perceived social support, which can lead to depression. The study has shown that individuals with ruminative thinking styles who are extremely negative when exposed to life events increase their risk of depression (Monroe and Simons, 1991; Nolen-Hoeksema and Morrow, 1991). The study found that individuals faced with many stressful life events and if they did not get enough social support, it would lead to depression (Guo et al., 2003). In other words, good social support is a good protective system that can mitigate the effects of stressful life events on depression (Broadhead et al., 2001). Social support has a buffer effect on stressful events, and rumination, which is a bad style of doing things, will aggravate adolescents’ psychological stress. High level of social support may alleviate adolescents’ psychological inappropriateness and then reduce adolescents’ active cognitive processing of traumatic events (Keppel-Benson et al., 2002). The present model shows that life events influence depression through the independent and combined effects of two mediating variables, namely rumination and perceived social support, which further support the mechanism of the influence of life events on depression. Understanding the influence of life events on adolescent depression and the underlying psychological mechanism is very important for the prevention and treatment of adolescent depression.

## Conclusion

Life events positively predicted the occurrence of depression and demonstrated that rumination and perceived social support played a mediating role in the relationship between life events and depression in middle school students. Specifically, more negative life events predicted stronger rumination and lower perceived social support, leading to the occurrence of depression. The results of this study not only provide new directions for the relationship between life events and depression, but also provide possible approaches for future prevention and intervention of depression in middle school students.

## Limitations

There are several limitations in this study. First, this survey was based on cross-sectional design, and convenience sampling method was adopted to select samples. It may be meaningless to generalize the survey results of the population samples to the population, and no causal conclusion can be inferred. Second, the small number of selected samples and the relatively narrow geographical coverage may lead to incomplete results. Third, all scales were used as online questionnaires, which would lead to low reliability of the data. However, it was important to realize that our model can still be extended and that there were other mediating variables.

## Data availability statement

The original contributions presented in this study are included in the article/supplementary material, further inquiries can be directed to the corresponding author/s.

## Ethics statement

The studies involving human participants were reviewed and approved by Research Ethics Committee in Jining Medical University. Written informed consent to participate in this study was provided by the participants or theri legal guardian/next of kin. Written informed consent was obtained from the individual(s), and minor(s)’ legal guardian/next of kin, for the publication of any potentially identifiable images or data included in this article.

## Author contributions

YL and YW contributed to the study design. JC and XH did literature search and screening. HX and DL independently extracted data. HX and XH analyzed the data and wrote this manuscript. YL and YW did the English revision. All authors reviewed the manuscript.
